# Implementing FAIR data management within the German Network for Bioinformatics Infrastructure (de.NBI) exemplified by selected use cases

**DOI:** 10.1093/bib/bbab010

**Published:** 2021-02-16

**Authors:** Gerhard Mayer, Wolfgang Müller, Karin Schork, Julian Uszkoreit, Andreas Weidemann, Ulrike Wittig, Maja Rey, Christian Quast, Janine Felden, Frank Oliver Glöckner, Matthias Lange, Daniel Arend, Sebastian Beier, Astrid Junker, Uwe Scholz, Danuta Schüler, Hans A Kestler, Daniel Wibberg, Alfred Pühler, Sven Twardziok, Jürgen Eils, Roland Eils, Steve Hoffmann, Martin Eisenacher, Michael Turewicz

**Affiliations:** Ruhr University Bochum, Faculty of Medicine, Medizinisches Proteom-Center, Bochum, Germany; Ruhr University Bochum, Center for Protein Diagnostics (ProDi), Medical Proteome Analysis, Bochum, Germany; Ulm University, Institute of Medical Systems Biology, Ulm, Germany; Heidelberg Institute for Theoretical Studies (HITS gGmbH), Scientific Databases and Visualization Group, Heidelberg, Germany; Ruhr University Bochum, Faculty of Medicine, Medizinisches Proteom-Center, Bochum, Germany; Ruhr University Bochum, Center for Protein Diagnostics (ProDi), Medical Proteome Analysis, Bochum, Germany; Ruhr University Bochum, Faculty of Medicine, Medizinisches Proteom-Center, Bochum, Germany; Ruhr University Bochum, Center for Protein Diagnostics (ProDi), Medical Proteome Analysis, Bochum, Germany; Heidelberg Institute for Theoretical Studies (HITS gGmbH), Scientific Databases and Visualization Group, Heidelberg, Germany; Heidelberg Institute for Theoretical Studies (HITS gGmbH), Scientific Databases and Visualization Group, Heidelberg, Germany; Heidelberg Institute for Theoretical Studies (HITS gGmbH), Scientific Databases and Visualization Group, Heidelberg, Germany; Jacobs University Bremen gGmbH, Bremen, Germany; Jacobs University Bremen gGmbH, Bremen, Germany; University of Bremen, MARUM - Center for Marine Environmental Sciences, Bremen, Germany; Jacobs University Bremen gGmbH, Bremen, Germany; University of Bremen, MARUM - Center for Marine Environmental Sciences, Bremen, Germany; Alfred Wegener Institute - Helmholtz Center for Polar- and Marine Research, Bremerhaven, Germany; Leibniz Institute of Plant Genetics and Crop Plant Research (IPK) Gatersleben, Seeland, Germany; Leibniz Institute of Plant Genetics and Crop Plant Research (IPK) Gatersleben, Seeland, Germany; Leibniz Institute of Plant Genetics and Crop Plant Research (IPK) Gatersleben, Seeland, Germany; Leibniz Institute of Plant Genetics and Crop Plant Research (IPK) Gatersleben, Seeland, Germany; Leibniz Institute of Plant Genetics and Crop Plant Research (IPK) Gatersleben, Seeland, Germany; Leibniz Institute of Plant Genetics and Crop Plant Research (IPK) Gatersleben, Seeland, Germany; Ulm University, Institute of Medical Systems Biology, Ulm, Germany; Leibniz Institute on Ageing - Fritz Lipmann Institute, Jena; Bielefeld University, Center for Biotechnology (CeBiTec), Bielefeld, Germany; Bielefeld University, Center for Biotechnology (CeBiTec), Bielefeld, Germany; Charité - Universitätsmedizin Berlin, corporate member of Freie Universität Berlin, Humboldt-Universität zu Berlin, and Berlin Institute of Health (BIH), Center for Digital Health, Berlin, Germany; Charité - Universitätsmedizin Berlin, corporate member of Freie Universität Berlin, Humboldt-Universität zu Berlin, and Berlin Institute of Health (BIH), Center for Digital Health, Berlin, Germany; Charité - Universitätsmedizin Berlin, corporate member of Freie Universität Berlin, Humboldt-Universität zu Berlin, and Berlin Institute of Health (BIH), Center for Digital Health, Berlin, Germany; Heidelberg University Hospital and BioQuant, Health Data Science Unit, Heidelberg, Germany; Leibniz Institute on Ageing - Fritz Lipmann Institute, Jena; Ruhr University Bochum, Faculty of Medicine, Medizinisches Proteom-Center, Bochum, Germany; Ruhr University Bochum, Center for Protein Diagnostics (ProDi), Medical Proteome Analysis, Bochum, Germany; Ruhr University Bochum, Faculty of Medicine, Medizinisches Proteom-Center, Bochum, Germany; Ruhr University Bochum, Center for Protein Diagnostics (ProDi), Medical Proteome Analysis, Bochum, Germany

**Keywords:** data management, de.NBI, FAIR principles, hourglass model, self-assessment, data maturity

## Abstract

This article describes some use case studies and self-assessments of FAIR status of de.NBI services to illustrate the challenges and requirements for the definition of the needs of adhering to the FAIR (findable, accessible, interoperable and reusable) data principles in a large distributed bioinformatics infrastructure. We address the challenge of heterogeneity of wet lab technologies, data, metadata, software, computational workflows and the levels of implementation and monitoring of FAIR principles within the different bioinformatics sub-disciplines joint in de.NBI. On the one hand, this broad service landscape and the excellent network of experts are a strong basis for the development of useful research data management plans. On the other hand, the large number of tools and techniques maintained by distributed teams renders FAIR compliance challenging.

## Introduction

Historically, the technical aspects of data management like data modelling, database technology, storage management, data integrity as well as the management of backup/archiving and recovery have been of utmost importance in life science [1]. Data repositories, in which the research datasets are stored at the end of an investigation, ensure the long-term storage and handle these aspects. Within this paper, we investigate this topic in the context of the German Network for Bioinformatics Infrastructure (de.NBI) [2] that is the basis for the German node of ELIXIR, the European bioinformatics infrastructure network.

Some journals require the deposition of the data underlying a research paper into public data repositories [3]. Many of them are very data domain specific and widely accepted by their community such as the ENA [4] and other INSDC [5] databases for genomics, PRIDE [6] for proteomics or the BioModels Database [7] for systems biology models. Also more general platforms like Dryad [8] or Zenodo [9] are widely accepted by publishers and therefore very popular within the research community.

While the mentioned repositories are accepted ‘endpoints’ of research data, they don’t map *the* ‘ongoing’ research, each are specialized on a relatively small target ‘domain’*,* such that typical projects need ‘combinations of services’*,* as well as ‘combinations of repositories’ to store their data*.* This puts data management early on in the research process into focus. It is recommended to start planning for data management already during the planning phase of a research project. That is the reason why funders of research and infrastructure projects require a detailed data management plan already as a part of grant proposals today [10].

With the emergence of open science and open data, organizational and data descriptive aspects that encourage data sharing and reuse are becoming more important. Therefore, the description of data by metadata in order to facilitate the retrieval of and access to such data and enable data integration, e.g. in multi-omics studies, is a key requirement. Additionally, interoperable data and tools allow for the automatic creation of workflows [11] and the reuse of data, either for reproducibility or for data reanalysis in view of new research questions. This requires well-annotated data and tools. For that purpose, de.NBI has registered most of the tools it offers, divided into subdomains, in the bio.tools registry [12] using terms from the EDAM ontology [13,14] to specify the supported input and output formats and other characteristics of these tools.

Key data management requirements are defined by the FAIR (findable, accessible, interoperable and reusable) guiding principles (Wilkinson et al. [15]). A framework of templates for defining metrics that measure the degree of compliance with these FAIR principles has been also published [16]. Since the data protection laws led to additional requirements for data privacy and data security, such requirements were included into the FAIR-Health principles [17], a proposal to extend the Wilkinson FAIR principles. This FAIR-Health proposal also contains additional requirements for information on the sample material used from biobanks, for provenance information and for incentive schemes. For cases where such privacy, ethical and legal requirements are important, e.g. in clinical studies underlying regulatory requirements, Woolley et al. described ADA-M, a matrix model for capturing and communicating metadata in a standardized way [18].

de.NBI [2] is a large service provider in the domain of bioinformatics. Its Special Interest Group ‘SIG4–Interoperability and Data Management’ aims at ‘facilitating FAIRness for the service users of de.NBI’*.* Ideally, a de.NBI user can obtain FAIR data without additional work. We aim at a ‘concept’ that is not a classical data management plan but rather a blueprint for creating bespoke data management plans from de.NBI services. Within this paper we present selected use cases within de.NBI that form a basis of this concept and are relevant for a broad audience within the bioinformatics community. These use cases as well as the subsequent discussion of more general aspects can be considered as a set of recommendations for all providers of similar bioinformatics tools, pipelines and other services, who are going to implement, assess and/or improve the fulfillment of FAIR criteria.

## FAIR data management landscape

In the past decade, we have seen a rise in the awareness that research infrastructure is important for the success of research. This has led to establishment of research infrastructures such as Europe’s ESFRI infrastructure ELIXIR and the German BMBF-funded de.NBI. They act in an ecosystem of FOSS and proprietary tools, as well as a large number of community standardization bodies, such as HUPO-PSI, DIVSEEK or COMBINE, and international organizations such as the Research Data Alliance, the GO-FAIR initiative and the Global Alliance for Genomics and Health, GA4GH.

Within the field of life science there is a huge number of tools, repositories and standards. FAIRsharing [19] provides an overview (see https://fairsharing.org). Organizations such as GO-FAIR suggest learning from the Internet’s ‘Hourglass Model’ that is seen as a key for its success [20]: a large number of user-level protocols and a number of hardware-level protocols were connected by just one core protocol: IP.

While few people think that just one tool will be ‘it’, there are two types of initiatives in the life sciences that follow the hourglass models: There are standardization efforts that facilitate interfacing tools, and there are efforts work in the direction ‘combinations’ of tools, such as the ELIXIR CONVERGE project.

The directions taken within de.NBI are part of this trend. Numerous de.NBI partners are active in standardization activities, and the present paper reflects our work towards pipelines of tools that go from data creation to data management system to publication.

As data management tools centers have a small number of solutions:

- The FAIRDOMHub that is based on the FOS software FAIRDOM SEEK [21,22]
- e!DAL-PGP for plant phenotyping data [23,24]
- PANGAEA for environmental and biodiversity data [25]

In addition, there is an ecosystem of related tools, standards and projects, such as GFBio and FAIRDOM. There are diverse approaches to metadata collection and use. Details are presented in the corresponding use cases.

These use cases across de.NBI service centers were chosen as examples, to illustrate the maturation of the FAIR criteria and the different classes of services. They highlight both challenges and recommendations that exemplify the transition of services towards FAIR criteria compliance. At the same time, they also show the diversity of implementation strategies for the FAIR indicators in a federated service landscape such as de.NBI. The de.NBI network supports this process by coordinating the implementation of the FAIR criteria through a managed self-organization approach. In particular, this approach focuses on the cataloguing of services, the standards used for metadata, data and formats, service metrics, the coordination of operations and the definition of overarching guidelines. This broad coordination in the community is especially important because de.NBI must take into account the federated character of its historically grown services.

The success of this concept is presented by exemplary use cases in the domains of proteomics, plant phenotyping and genotyping as well as human genomics in the section ‘use cases’. Here we show in particular, the benefits of agreements reached at the levels of harmonization of metadata, data formats and data publication infrastructures, which enabled the implementation and co-development of accepted standards for metadata, centrally managed and organized consulting and trainings and data publication pipelines into sustainably operated infrastructures. In order to integrate these into the actual institutional research data management (RDM) processes, a cultural change was required, which is now significantly supported by a broad acceptance of data as scientific and sustainable services for infrastructural assets in Germany’s life science landscape. This made it possible to implement a high quality and continuous research data and service management process from experimental design to data analysis and finally the re-use of data. This has been significantly catalysed by infrastructure networks such as de.NBI. Especially the integration of national and international activities through the establishment of the German ELIXIR node by de.NBI enabled joint training, outreach and linking of partners within collaborative research projects.

## Self-assessment of FAIR criteria

**Figure 1:**
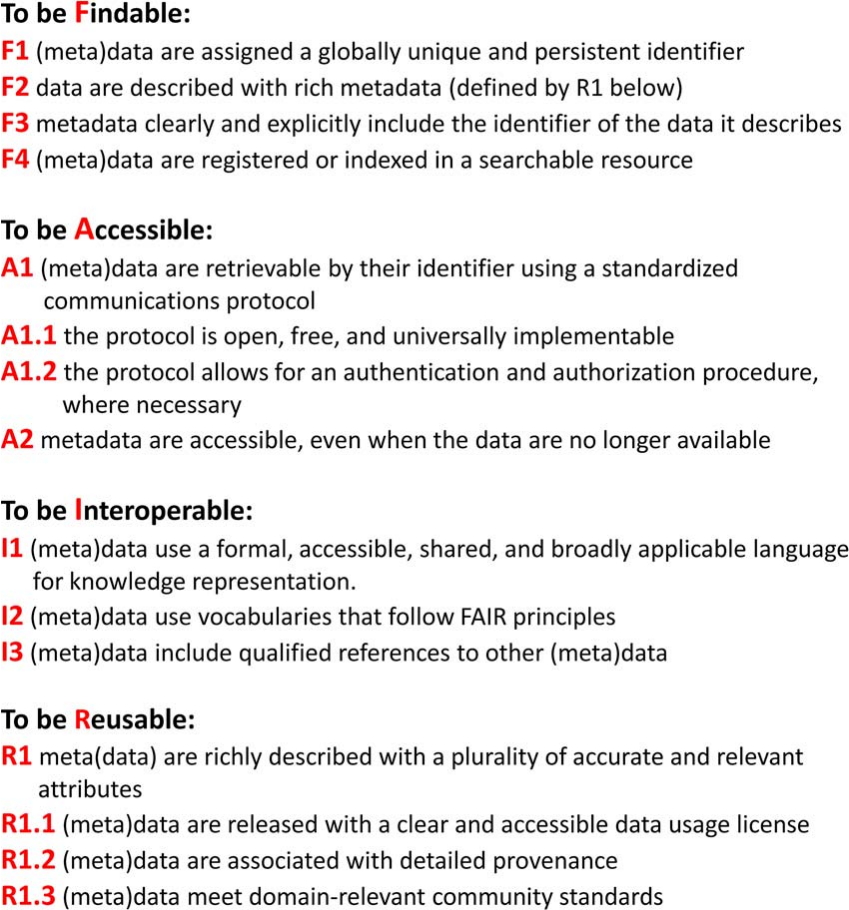
The FAIR guiding principles as formulated by Wilkinson et.al [15].

In order to fully implement a sustainable data management plan, data must be FAIR for both humans and machines. The FAIR guiding principles formulated by Wilkinson et al. [15] are shown in Figure 1.

While waiting for widely accepted FAIRness tests that cover metadata quality, the best practice is to self-assess compliance with the FAIR criteria. In the literature, self-assessment usually leads to a grouping into three different categories. Either the assessment concludes that the data partly, fully or not at all fulfil the FAIR criterion, although the degree of compliance may vary, especially in the case of ‘partial’ compliance. In general, only a small number of software is described in regards to its FAIR compliance in the literature. Out of more than 50 found references to the FAIR metrics publication [16] only seven actually described software (although sometimes more than one single software) and its FAIRness. However, repositories like FAIRsharing [19] list many more FAIR-compliant software instances.

Frameworks for the automated verification and assessment of FAIR conformity have recently experienced an increase in popularity. These use automatic test metrics to assess the different levels of FAIRness either in relation to the underlying data or the software itself. For this test setup to work properly, the resource must be web based, which is why offline resources cannot be evaluated with these services and rely on self-assessment.

## Use cases

We describe six selected use cases. Each of them describes the process of data processing and the enrichment of metadata up to the final data storage in archives or repositories. Special attention is paid to the ‘first mile’, where essential local data are created and captured first, and the hard work of building modern data-pipelines just begins. The endpoint for our view on these use cases is the so-called ‘last mile’, which in accordance to ‘first mile’ references the deposition of primary data and associated metadata to appropriate long term storage repositories. Relevant process steps are evaluated as self-assessment according to the fulfilment of the FAIR metrics [16]. For this purpose, the 13 metrics with 3 classes of the degree of fulfilment are evaluated with ‘Yes’, ‘No’ and ‘Partly’. The proof of the assessment is explained in the text.

### Use case 1: bioinformatics and statistical consulting

The de.NBI service center BioInfra.Prot [26] provides a service for bioinformatics and/or statistical consulting and analysis of quantitative proteomics data. The actual analysis is tailored to the specific needs of the user. Basically, there are two different types of metadata for this service. First, there is the metadata that the user provides and that is needed to conduct the analysis. This includes, for example, information regarding the experimental groups or already performed preprocessing steps applied to the data. During the execution and documentation of the analysis, the second type of metadata is generated by the service. It contains, e.g. detailed information about the statistical or machine learning methods performed. Unfortunately, due to the large variety of possible methods and experimental designs, there are currently no widely accepted guidelines on the minimum information needed to describe such statistical and/or bioinformatics analysis, although efforts are already being made in the fields of metabolomics and lipidomics [27,28].

**Figure 2:**
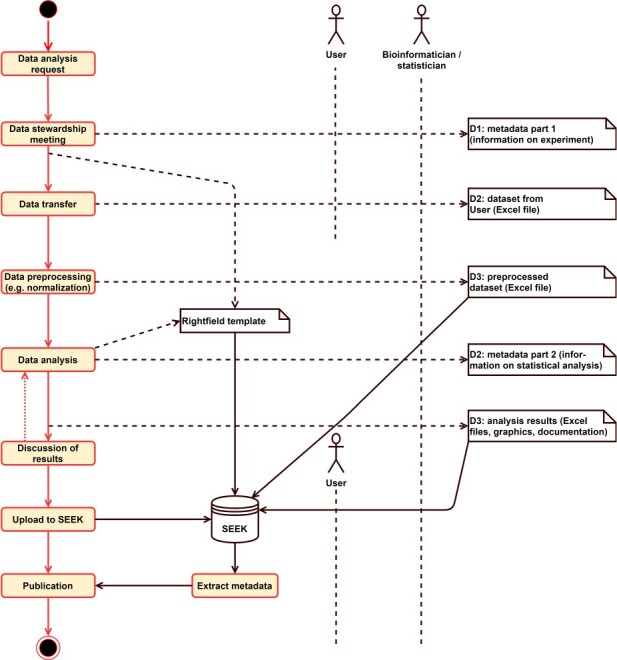
Bioinformatics and statistical consulting workflow, showing the collection of metadata in two parts using a RightField template. Together with the analysis results the metadata is stored in SEEK and directly usable for the publication process.

For documenting the results of the bioinformatics and statistics analyses, we use the ontologies STATO (http://stato-ontology.org) and OBCS (Ontology of Biological and Clinical Statistics) [29]. Furthermore, we employ the FAIRDOM RightField tool [30] to create Excel templates that contain the possible controlled vocabulary (CV) terms given in these ontologies as allowed values in spreadsheet cells. This Excel file consists of two worksheets, one for each of the two types of metadata. The first worksheet is completed together with the user of the consulting service during a data stewardship meeting, where the aims of the study and the required analyses are discussed and defined with the user. This is the first mile of this use case. Then, the scientist conducting the service fills in the second worksheet while the analysis is being carried out. These collected metadata complement the resulting tables and figures and are useful for the documentation of the analyses and for writing the methods section of the associated publication or thesis. Furthermore, the Excel file with the captured metadata will be uploaded together with the other result files to SEEK [21]/FAIRDOMHub [22], which then assigns a unique identifier to the whole study dataset. This is the last mile of this use case. Figure 2 summarizes the complete workflow.

Table 1 shows how the FAIRness of the data from this use case is enhanced by capturing and documenting the metadata in Excel templates defined by RightField. Summarizing, in this use case, we use Excel spreadsheets that are easy to set up and also easy to fill out for the scientists. Normally, to achieve a similar level of user-friendliness, the expensive development of a user interface would be necessary, which we avoid by using RightField. By using RightField-Enabled Excel templates, information can be entered using closed vocabulary selection lists, reducing errors. The resulting data then can easily be managed using the FAIRDOMHub. Thus, the use case demonstrates how combination of simple, generic tools can lower the bar towards FAIR data management. The method can be applied in all use cases that concern table-based data that have to be entered or completed by humans.

**Table 1:** Self-assessed degree of fulfilment of the FAIR criteria for a bioinformatics resp. statistical consulting and analysis. Case (1) without and case (2) with the use of metadata captured in RightField templates and uploaded to SEEK/FAIRDOMHub

	F1	F2	F3	F4	A1	A2	I1	I2	I3	R1.1	R1.2	R1.3
Without metadata	No	Partly	No	No	No	No	No	No	No	No	Partly	No
With metadata	Yes	Yes	Yes	Yes	Partly	Partly	Partly	Partly	Partly	Partly	Yes	No

### Use case 2: PRIDE upload of proteomics data

Another service of BioInfra.Prot is the curation of proteomics dataset uploads to the public proteomics data repository PRIDE [6]. These uploads are performed by dataset submitters using the ProteomeXchange submission tool [31], which interactively requests some metadata describing the dataset. This step is the first mile of this use case. Some of these metadata are plain text fields like e.g. the project title, the project description as well as sample processing and data processing protocols. Others are CV terms from different ontologies, describing e.g. the type of proteomics experiment, the species, the tissue, the instrument used, the disease and the modifications involved in the peptide identification search. All this metadata collected by the ProteomeXchange submission tool is then written to a text-based file summarizing the submission [31].

In case of a complete submission, i.e. a submission, where the data are uploaded in standardized proteomics XML data formats like mzML [32,33] or mzIdentML [34], additional metadata is contained in <cvParam> elements within these files. These elements semantically annotate the respective XML element to which they belong by referencing a CV term from the psi-ms.obo ontology [35].

**Figure 3:**
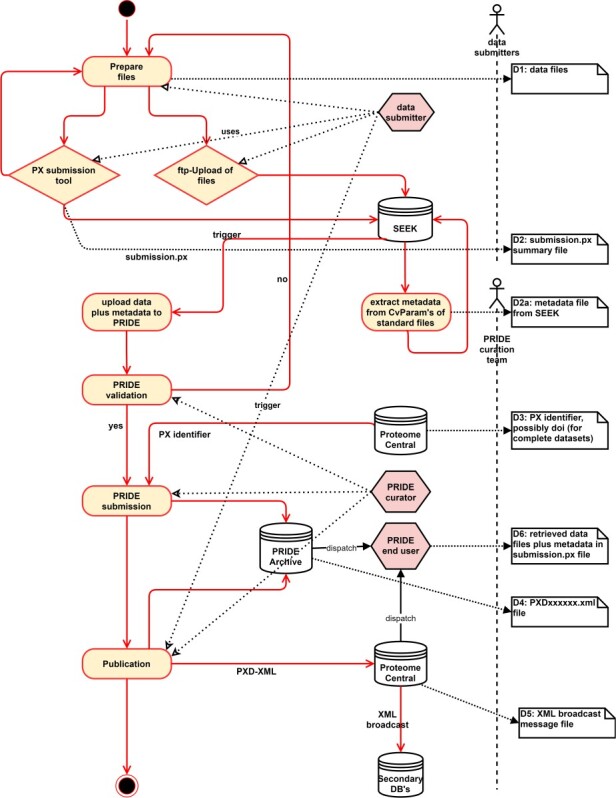
Upload of proteomics files in a standard format to SEEK and enrichment with metadata annotation extracted from these files. For the future it can be envisioned that the automatic upload from SEEK to PRIDE can be triggered by SEEK.

From files in proteomics standard formats, we read out all <cvParam> elements by using the xxindex (https://github.com/PRIDE-Utilities/xxindex) library. It allows one to read all CV terms and their corresponding values if values are assigned. Then, as the last mile of this use case, all found CV terms and their corresponding values are written into Excel sheets, which can be uploaded to SEEK together with the submission summary file and the data files. These Excel files containing the extracted metadata are machine-readable and may supplement PRIDE submissions in the future in order to complete the directly accessible metadata (Figure 3). In case of a partial submission, where the data are in proprietary data formats, as listed at http://wwwdev.ebi.ac.uk/pride/markdownpage/pridefileformats, only the data files and the submission summary file are uploaded to SEEK (Figure 3). Table 2 shows how the self-evaluated FAIRness of the PRIDE uploads use case.

**Table 2:** Self-assessed degree of fulfilment of the FAIR criteria for the PRIDE upload use case

F1	F2	F3	F4	A1	A2	I1	I2	I3	R1.1	R1.2	R1.3
Yes	Yes	Yes	Yes	Yes	No	Partly	Yes	Yes	Yes	Yes	Yes

This use case demonstrates the crucial role of well-defined standard data formats based on ontologies and CV terms. Unfortunately, in this use case, currently the users decide whether a complete submission based on standard data formats is performed. Thus, enforcing their usage by data repositories could ensure a more complete fulfillment of FAIR principles.

### Use case 3: PIA—protein inference algorithms

Protein inference algorithms (PIA) [36,37] is a toolbox for MS based protein inference and identification analysis. PIA allows the inspection of common proteomics spectrum identification search engine results, combine them seamlessly and conduct statistical analyses. The main focus of PIA lays on the integrated inference algorithms, i.e. concluding the proteins from a set of identified spectra.

**Figure 4:**
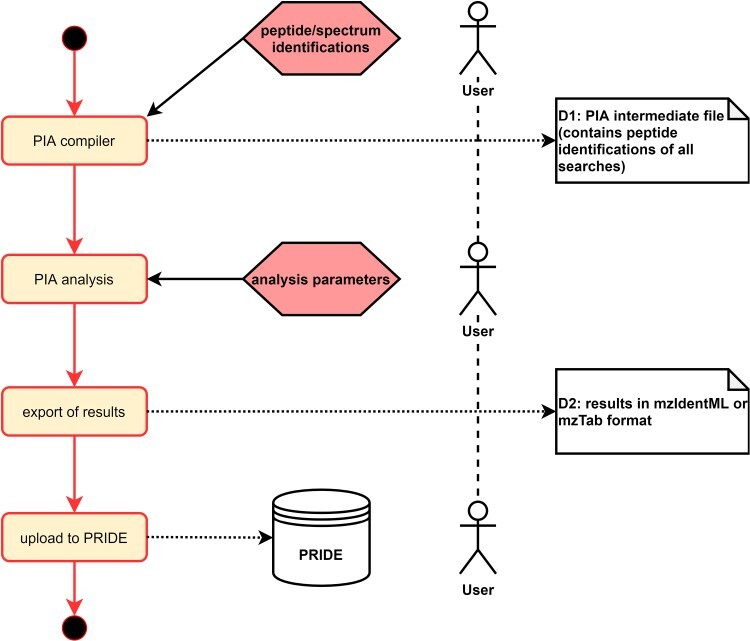
Flowchart diagram of a PIA analysis. The actual PIA analysis ends with the export of the results, preferably into one of the standard formats for protein identifications (mzIdentML or mzTab). The PRIDE upload is optional but is requested e.g. for a publication of the analysis.

The input for the tool can be any spectrum identification results provided in the mzIdentML format, which is enriched by metadata in XML <cvParam> elements as explained above. This is the first mile of this use case. The mzIdentML format is designed to explain in detail all steps of the analysis up to the currently performed step, including the entire data processing. In case of a PIA analysis, the spectral data itself (preferably in mzML format) is linked to the steps of spectrum identification with all parameters and any additional processing like the calculation of the false discovery rate with its settings. Finally, the selected options and parameters for protein inference are stored together with the peptide identifications and protein groups [38]. In case these options were used in the analysis, the same <cvParam> extraction method as described for the PRIDE upload use case is performed. The complete workflow is shown in Figure 4.

Unfortunately, as with almost all larger XML files, mzIdentML files are not easily parsed by post-processing steps and are also not suitable for human data inspection. Therefore, if an export of the PIA results into an easily editable format is preferred, they can be stored in mzTab [39] format. This format does not store all information needed to understand the analysis results. However, most common parameters are stored and mzTab can therefore be considered a compromise to the more comprehensive mzIdentML standard. The export of all metadata into PIA results files is the last mile of this use case.

Also here the benefit of standard file formats, which contain rich annotation of metadata for further processing, is demonstrated. To store the metadata in the metadata repository only the original PIA results files containing them in the <cvParam> elements (mzIdentML) or file header information (mzTab), respectively, must be uploaded. The self-assessment regarding the fulfilment of the FAIR criteria summarized in Table 3 shows that for this use case almost all criteria are fulfilled. This is because the upload of PIA output files to a searchable repository like PRIDE is optional. Consequently, F1 and F4 are considered only ‘partly’ fulfilled.

**Table 3:** Self-assessed degree of fulfilment of the FAIR criteria for the PIA use case

F1	F2	F3	F4	A1	A2	I1	I2	I3	R1.1	R1.2	R1.3
Partly	Yes	Yes	Partly	Yes	Yes	Yes	Yes	Yes	Yes	Yes	Yes

**Table 4:** Self-assessed degree of fulfillment of the FAIR criteria for the IPK NGS workflow use case

FM Step	F1	F2	F3	F4	A1	A2	I1	I2	I3	R1.1	R1.2	R1.3
Run config	Yes	Partly	No	Yes	Yes	Yes	No	No	Partly	No	No	No
Data archival	Yes	Partly	Partly	Yes	Yes	Yes	Partly	Partly	No	No	Partly	No
Upload to SRA	Yes	Yes	Yes	Yes	No	Yes	Yes	Partly	Partly	Yes	Partly	Yes

### Use case 4: integrated workflow for the handling of NGS data and metadata

**Figure 5:**
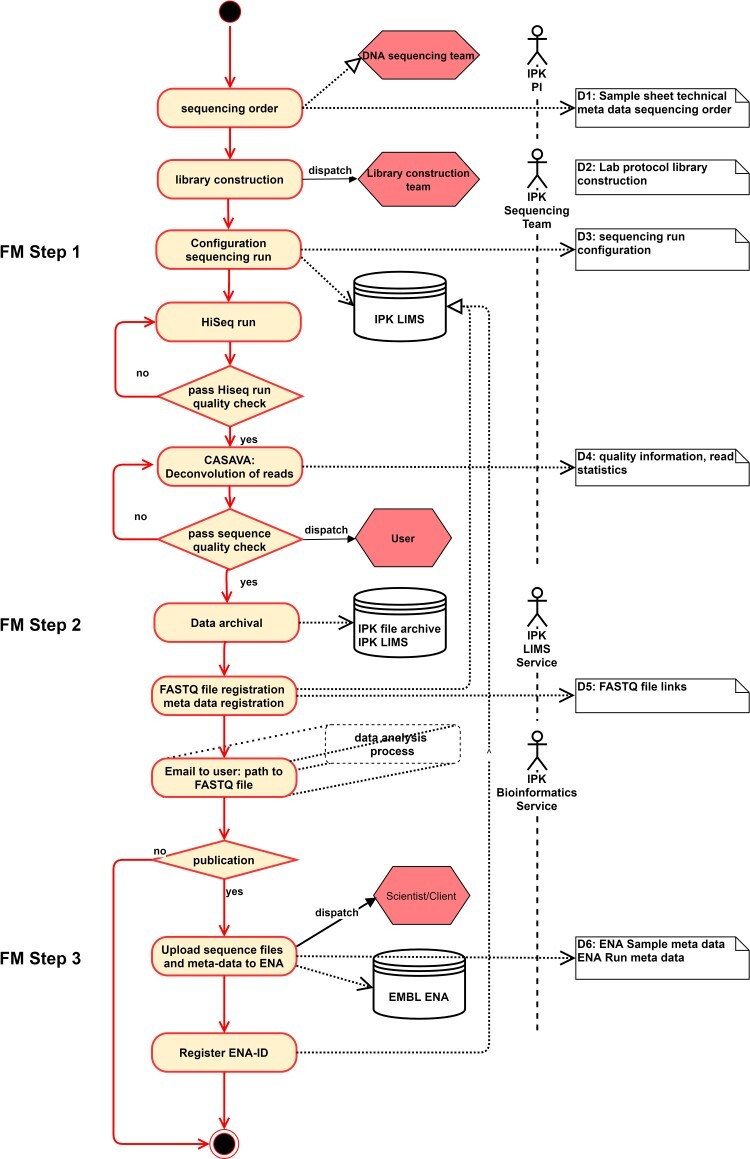
Flowchart diagram of export workflow to the European Nucleotide Archive (ENA).

The NGS data flow process executed at IPK Gatersleben, Germany, is illustrated in Figure 5. The process includes sequencing in the laboratory (step 1), transfer into the IPK Laboratory Information Management Systems (LIMS) as a generic data backend (step 2) and feeding into the EMBL-ENA repository [4] (step 3). The meta and sequence data are closely linked to each other and represents an enrichment over all process steps. This process is completely mapped in the central LIMS of the IPK, so that there are no data transfer points, only feed-in points. Since the FAIR quality of ENA archived sequences depends on the metadata quality of the individual process steps beforehand, we have evaluated the FAIRness of these three intermediate steps in the NGS process. Table 4 shows the results of this evaluation.

Step 1 is the first mile and crucial for the completeness of the metadata. Doing so a sequencing order is submitted to the sequencing laboratory as a sample form, which comprises contact information (e.g. client name, e-mail, billing information), sample description (e.g. species, sample name, sample type) and technical sequencing parameters (e.g. number of lanes, read length, type of library). These metadata are either entered or imported into the IPK LIMS by the customers or the sequencing staff. From this, a sequencing order and the necessary configuration files for the sequencing run are created including a unique numerical sample identifier, which is necessary for the later assignment of the sequence data.

In step 2, after the sequencing run and the subsequent base calling, the resulting FASTQ sequence data are demultiplexed and copied by a script to the IPK file server. The FASTQ files are named analog to the sample number and are permanently stored in folders named after the sequencing run ID. These storage paths are accumulated by the FASTQ distribution script in a CSV file and sent to the IPK LIMS manager. The latter imports these file links into the LIMS so that they are associated with the aforementioned sample information. Afterwards the completion of the sequencing order including the link to the FASTQ files and the sequencing order number is sent to the customer via e-mail.

Submission to the sequence read archive (SRA) of ENA in step 3 is the last mile and relies on metadata of step 1 as well as on personal feedback to the responsible scientists to clarify potential issues. This step is performed by a skilled data steward who finally curates the metadata and creates a so-called ‘BioProject’ or ‘Study’ describing the overall goal of an individual research endeavor. A BioProject may comprise multiple experiments submitted to different NCBI or ENA databases. Thus, the design of the sequence submission intrinsically promotes the aggregation of experiments that have been carried out to the same aim. Before the submission of sequence data, the SRA requires detailed descriptions of the ‘BioSamples’ from which the sequences were obtained. To facilitate the description and to improve the findability of BioSamples, the INSDC databases maintain structured attribute name-value pairs. The creation or registration of a BioProject and BioSamples is the prerequisite for registering an SRA experiment, which is the unique sequencing result of a specific sample and the last step before uploading individual sequencing ‘Runs’. For each registration step, the SRA database generates individual accession numbers with specific prefixes facilitating the findability of additional data generated in the course of the BioProject. A project may also contain other than just sequencing data; the metadata regimen facilitates the integration of various data types and experiments.

### Use case 5: human genomics data in the cloud

With growing amounts of human genomics data in the healthcare sector, sharing genomics data will be essential for research as well as routine diagnostics especially for treatment of cancer and rare diseases [40–42]. Thereby, genome data itself are sensitive personal data that need to be highly protected [43]. Having data sharing and data security in mind, several global initiatives such as the Global Alliance for Genomics and Health (GA4GH) are defining standards for management and processing of human genomic data. A basic concept is to send workflows to data and share only anonymized results instead of sharing data directly with other scientists or clinicians.

The Health-Data-Hub, a combined research group based at the Charité Universitätsmedizin Berlin, Berlin Institute of Health and Universität Heidelberg, aims to develop platforms specifically for sharing access to genomic data and other personal omics data from medical facilities. Such a platform needs to be based on open tools and standards. Having a platform hosted on an in house cloud, which is a part of the larger de.NBI cloud federation, supports having computing close to the storage of the genomic and medical data, which cannot be transferred easily for legal as well as technical reasons. Here, on the first mile, data providers manage data and provide indirect access to their data such as raw sequence files, alignment files or count matrices to other scientists and clinicians. Publication of descriptive metadata in public databases supports findability of their data and implementing confederated user authentication systems, such as ELIXIR AAI can provide a wide accessibility. On the last mile, once permitted, users can send verified workflows to the data and generate new results such as anonymized summary data. Building on GA4GH standards for workflow execution, data analysis is fully interoperable and reproducible while all workflow processing steps, used data sets and workflow configuration parameters are completely documented. Thus, the vast majority of FAIR criteria for both data storage and workflow execution are fulfilled or at least partly fulfilled in this use case (Table 5).

**Table 5:** Self-assessed degree of fulfilment of the FAIR criteria for the health-data-platform

Step	F1	F2	F3	F4	A1	A2	I1	I2	I3	R1.1	R1.2	R1.3
Data storage	Partly	Yes	Yes	Yes	Yes	No	Yes	Yes	Yes	Yes	Yes	Yes
Workflow execution	Partly	Yes	Yes	No	Yes	No	Partly	Partly	Partly	No	Yes	Yes

**Table 6:** Self-assessed degree of fulfilment of the FAIR criteria for the phenomics data management at IPK

F1	F2	F3	F4	A1	A2	I1	I2	I3	R1.1	R1.2	R1.3
Yes	Yes	Partly	Yes	Yes	Yes	Partly	Partly	Partly	Partly	Partly	Yes

### Use case 6: data life cycle for high throughput plant phenotyping in controlled environments

To tackle the challenge of FAIR documentation of phenomics experiments, the MIAPPE [44,45] consortium developed recommendations for a best-practice documentation. This minimum information standard serves as a framework to conceptualize the IPK data workflow. Beyond curated and standardized metadata, the publication of well-annotated datasets is important for community outreach. No dedicated plant phenomics-focused data repository exists hitherto; however, MIAPPE compliance is advertised and increasingly achieved by hosting data on-site and wrapping it in FAIR interfaces. IPK applies e!DAL-PGP infrastructure [24] and implements the BrAPI [46] specification for RESTful API to plant phenotyping data.

**Figure 6:**
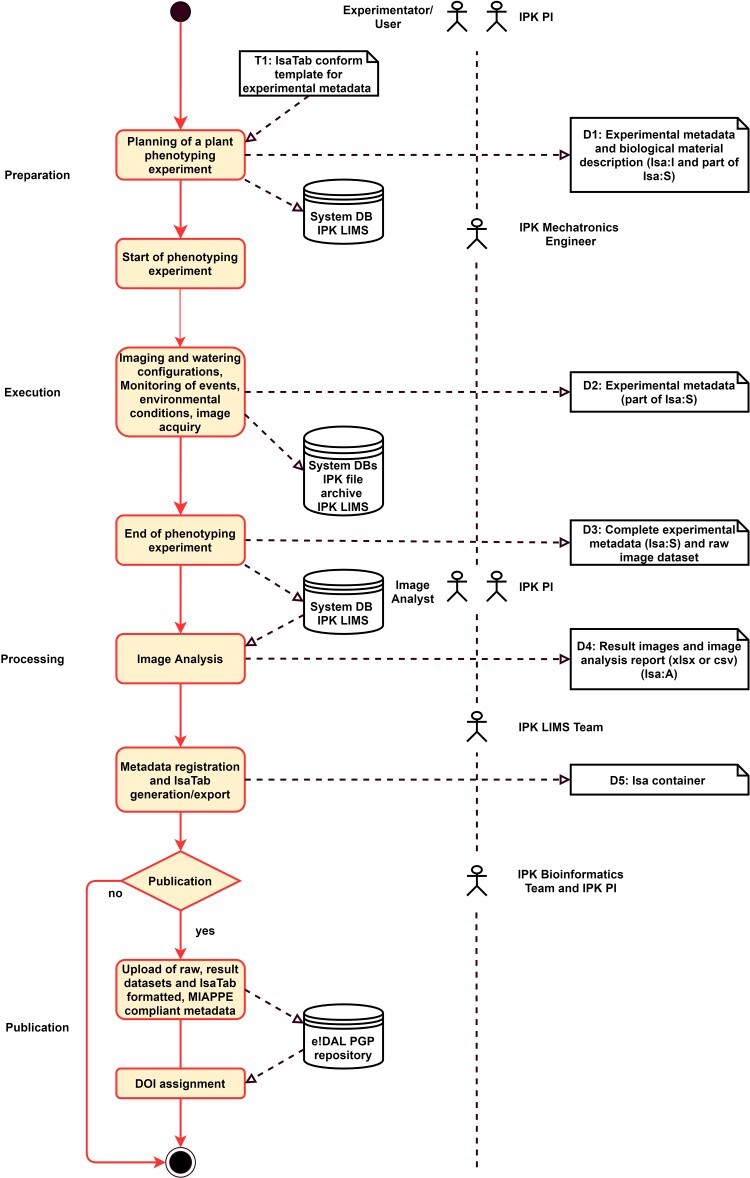
Flowchart diagram of phenomics data management at IPK.

The IPK houses a comprehensive research infrastructure for the quantitative assessment of whole plant features in controlled environment growth facilities [47]. The interdisciplinary phenotyping workflow is characterized by a complex interplay between gardeners, biologists, mechatronics engineers and IT specialists. A similar complexity is on the technical level of the multi-sensor systems combined with vendor specific embedded databases, which requires data conversions and interface wrapping to ingest into IPK data infrastructure and data processing pipelines. Currently a number of manual steps of (meta)data conversion, mapping and copying of scripts at the control PCs of the various systems are performed. Acquired images are transferred to the IPK hierarchical storage management system. The experimental set-up and linked metadata are imported into the IPK LIMS. The complex process of FAIR phenomics data management at IPK, which is illustrated in a flow process diagram in Figure 6, combines several automated steps, but still needs some manual processing, whereas standardized formats are used in all instances. All FAIR criteria are at least partly fulfilled in this use case (Table 6).

On the first mile, the setup of the phenotyping experiment is defined according to the platform capacity and the biological question of interest. A subset of MIAPPE-compliant attributes like plant IDs, experimental design and seeding date are filled into an ISA-structured template [48], which is uploaded into the vendor specific control software of the phenotyping devices and the IPK LIMS.

During the execution of the experiment, imaging and watering configurations are recorded in the embedded database of the phenotyping facility, whereas irregular events such as system error, transplanting events or manual measurements and fertilizer treatments are captured in the LIMS. Environmental and soil monitoring data, acquired through sensor networks, are delivered using the MQTT protocol. Because of the high number of sensor messages, these data are recorded in specifically designed and compressed database tables. At the end of the experiment, the metadata is curated and enriched by MIAPPE study level information.

The last mile is characterized by transferring the raw image data into IPK central storage management system. Beyond this, the images can be fed into the IPK image analysis pipeline. Result images and tabular report files are later registered in the LIMS. In order to publish a combined set of raw images, edited images and extracted features, such as architectural and biomass-related traits, a data container is manually compiled. The extracted measurements and metadata are encoded as ISA-TAB files. The complete dataset can be finally published in the mentioned e!DAL PGP repository. The assigned DOI can be cited in data publications [24] or biological papers [49].

## Discussion

### What we have learned?

As shown for use case 1, even for a data analysis and consulting service all FAIR principles can be completely or partly fulfilled, when applied to metadata. Moreover, the described mechanism employing RightField and SEEK allows the fulfillment of FAIR principles for a wide range of use cases dealing with spreadsheet data. These tools are free, versatile and not difficult to establish and provide a good cost–benefit ratio. Thus, we recommend them for similar services, especially, for academia and other service providers with few financial resources for data management.

In use cases 2 and 3 the benefits of well-annotated standard data formats based on standard ontologies and controlled vocabularies were demonstrated for a data publication service and a software tool. Thus, we recommend the exclusive usage of such standard data formats. However, as described in use case 2, often the users decide whether standard data formats are employed since data repositories usually allow data submissions with non-standard formats. In order to address this general issue, in our opinion, both should be pursued educational work among service users and community-driven standardization efforts among software developers. Both are long-term processes. Their goal should be to increase the usage and the general benefits of standard formats.

Careful documentation and maintenance of all metadata information throughout the life cycle of entire work processes, such as in the above-mentioned use cases 4 and 6, can not only facilitate the publication of research data but also provide the opportunity to reuse data, as aimed at by the FAIR principles. Experience shows that metadata that has to be added shortly before publication and not already at the time of data generation can often no longer be fully comprehensible, so that the quality is significantly lower. We recommend that this should be avoided by being as precise as possible from start to finish of an experiment and not neglect the metadata. Furthermore it is important to integrate standardized interfaces between the different process stages whenever possible and to avoid manual curation steps.

### General aspects

Since many different bioinformatics infrastructures are provided within de.NBI for almost all life science disciplines, there are many different kinds of research data, file formats and workflows that are managed by the different de.NBI sites. Consequently, there are several heterogeneous data management use cases demonstrating that it is challenging to formulate general data management concepts for heterogeneous consortia such as de.NBI or ELIXIR.

The described use cases reflect the broad range of de.NBI service categories including offline software tools and pipelines as well as services with manual or semi-manual data processing steps, which are frequently located at the very first mile of data processing. Automatic assessment frameworks and tools like FAIR Evaluator and FAIRshake [50,51], which employ a set of FAIR metrics or maturity indicators [16], were implemented to evaluate online data resources, which are a minority among de.NBI services. Consequently, these frameworks are unsuitable for general evaluation guidelines within our data management concept. For the vast number of our services self-assessment is the most obvious approach since dedicated FAIR assessment teams or crowdsourcing strategies would be less effective as evaluation by service maintainers and would require additional personal resources. Thus, we recommend self-assessment as the best approach for service providers with low resources and services, which cannot be evaluated by automatic assessment frameworks. However, as the evolution of automatic evaluation frameworks proceeds, they may be more generally applicable in the future.

In order to facilitate self-assessment and the improvement of FAIR data management, we recommend that the maintainers of bioinformatics services receive further training in data stewardship. This is increasingly supported by the free training activities provided by de.NBI. Moreover, some de.NBI members are partners in the ELIXIR CONVERGE project, which creates a network of data management experts for collaboration and knowledge exchange between domain experts. A main focus is on the development of an RDM toolkit to synchronize and standardize data management activities in Europe and to help researchers in improving the FAIRness of their data. We recommend using this infrastructure in the future.

We imagine RDM as a process from cradle to data publication, starting with Electronic Lab Notebooks or LIMS at the lab bench, use of bioinformatics tools for analysis and then feeding into an RDM system like e.g. SEEK or e!DAL for further processing. Once the processing of the data is complete, they can be stored together with the result files in specific domain repositories like PRIDE or ENA or general data publication platforms like Zenodo, where they are made publicly available after publication of the corresponding paper.

There are various useful tools that facilitate the implementation of FAIR data management strategies. For example, RightField [30] creates Excel files that are primarily designed for interactive metadata acquisition and can be employed in all use cases where metadata have to be entered or completed by humans. Furthermore, it is possible to employ SEEK, another useful software, for collecting the metadata together with all data files and then trigger an automatic upload of these data along with the associated metadata into the appropriate data repository, e.g. PRIDE for a proteomics submission [52]. Another solution would be the use of the e!DAL software infrastructure as on-premise infrastructure for managing and describing diverse types of research data during the research process and for sharing them at the end with the community by assigning a DOI and reference them in an associated article or a data paper. This is already practised with the e!DAL-PGP repository at IPK and a further e!DAL-based infrastructure at FZJ. Beside these systems, which are hosted and maintained by de.NBI partners, there are also other software infrastructure available like CKAN or DataVerse that are providing a similar functionality and have a growing number of international users. We recommend using such free, versatile and straightforward tools that facilitate FAIR data management and provide a very good cost–benefit ratio. To find these tools we recommend FAIR tool catalogues such as the RDM toolkit (https://rdm.elixir-europe.org) currently developed within the ELIXIR CONVERGE project.

To date, we have no clinical use cases where data privacy, data security and/or ethical concerns play a role. For implementing such use cases an additional access control to the data is required. We plan to extend our de.NBI data management concept to such use cases in the future, e.g. by including the FAIR-Health principles [17].

In general, to improve data management, in the first step, we recommend implementing measures that are free and as simple as possible. This includes the increased use of free data management software and standard data formats, as well as the further training of already employed tool maintainers in FAIR data management. An increasing compliance with the FAIR criteria should be used as a measure of success. At least partial fulfillment of all criteria should be the minimum goal. This first step should also be feasible for service providers with few financial resources. Only in the second step should the extensive use of professional data stewards and proprietary or commercial software tools be considered for complete fulfillment of all FAIR criteria. Even if the second step is only feasible for service providers with extensive funding for data management, in our experience the implementation of the first step already significantly improves the fulfillment of the FAIR criteria.

### FAIR for research software

As described, not all FAIR principles can be completely fulfilled for our use cases resulting in some ‘Partly’ and ‘No’ entries in our self-assessment tables. In this context, it is questionable whether all categories of services such as software tools or analysis pipelines can generally fulfill all original FAIR principles.

Data can be regarded as any digital information including both factual information and computer instructions of software and workflows. While factual information is both editable and readable, computer instructions can also be executed. Consequently, it is questionable to what extent the FAIR principles also apply to software.

Recently, Lamprecht et al. [53] argued that most of the FAIR principles can be easily adapted to software with a few minor modifications. Particularly software is in a constant state of change caused by updates and improvements. The appropriate management of all software dependencies must be documented with rich metadata. Therefore a long-term stable versioning and indexing of software versions are necessary to make the software FAIR. Moreover, since further development of operating systems and dependencies makes the long-term sustainability of scientific software extremely challenging, we recommend employing virtualization, containerization with frameworks such as BioContainers [54] and package management with platforms such as Bioconda [55] to address this issue. However, to enable users to use containerized software and package managers, specific user training and additional documentation are needed. A critical point is the functional correctness of software, which goes far beyond the current FAIR principles. It can be argued that meaningful metrics must first be established in order to formulate FAIR-compliant functional correctness principles. Moreover, while the FAIR principles do not require data to be open, in most cases openness can be expected for research software [56].

### Training activities

To complete our data management-related activities, de.NBI provides training for data management [57] in order to sensitize users for the benefits of FAIR data management and to educate them in best practices. To improve FAIR awareness, training is crucial because in our experience based on user discussions during various training events on other topics still too many users have little knowledge about the advantages of FAIR data management and, consequently, have no FAIR awareness. Therefore, some de.NBI training events related to FAIR data management were advertised and conducted to close these gaps for at least some of our users. In total, more than 300 participants (as of September 2020) have been trained in 13 data management training courses since 2015. These courses are mainly organized by the service centers BioData (providing the widely used data resources SILVA [58], PANGAEA [25], BacDive [59], BRENDA [60] and ProteinsPlus [61]), GCBN (providing services and data infrastructures such as e!DAL-PGP repository [23,24], PlantsDB [62] and Trimmomatic [63]) and de.NBI-SysBio (providing data management-related tools like SEEK [21] and on-site visits on request to support customers in installing their own data management projects or local SEEK instance). In the future, de.NBI intends to further increase the amount of training courses in the fields of data management and FAIR data. Another step towards training scientists in data management will be the initiation of a de.NBI data stewardship program, which will be similar to the FAIR data stewardship program organized by the Dutch TechCentre for Life Sciences in the Netherlands (https://www.dtls.nl/fair-data/). Data stewards are persons that have specific technical (i.e. experience in metadata, software tools, workflows and programming) and communication skills (i.e. communication with life scientists, data producers and data analysts), which are needed to implement professional data management. de.NBI is involved in different initiatives to develop a curriculum for data stewardship and data management within Germany and Europe (e.g. Project 29 at the BioHackathon 2020: ‘Design of a modular learning path (curriculum) in Data Stewardship, Management and Analysis for the Life Sciences’). In our experience, besides usual training courses, additional measures are needed. Hence, we recommend a close interconnection of a training program with user support and consulting, since, in our experience, this works best to motivate users to start implementing a FAIR data management.

**Key points**

- Description of six data management use cases as basis for derivation of guidelines for a FAIR data management concept within a large bioinformatics infrastructure network such as de.NBI.
- Description of the metadata capturing and data management process for one consulting and two repository upload activities demonstrating that FAIR data management is basically possible for this kind of services.
- Self-assessment of the degree of fulfilment of the FAIR criteria facilitates the development and comparison of data management concepts for service categories where automatic assessment tools cannot yet be employed such as consulting services.
- The benefits of standard data formats and data repositories were demonstrated. However, users often decide whether data in standard data formats are published in repositories. In order to improve FAIRness it would be advantageous if repositories would enforce the usage of standard data formats.
- In order to sensitize users for the benefits of FAIR data management and to educate them in best practices user training is crucial.

## Notes

**Gerhard Mayer** is a research associate who worked at the Medizinisches Proteom-Center of the Ruhr University Bochum and works now at the Institute of Medical Systems Biology of Ulm University.

**Wolfgang Müller** is a group leader for Scientific Databases and Visualization at the Heidelberg Institute for Theoretical Studies.

**Karin Schork**: is a statistician and a PhD student in the research unit Medical Bioinformatics at the Medizinisches Proteom-Center within the Medical Faculty of the Ruhr University Bochum.

**Julian Uszkoreit** is a postdoc in the research unit Medical Bioinformatics at the Medizinisches Proteom-Center within the Medical Faculty of the Ruhr University Bochum.

**Andreas Weidemann** is a postdoc in the group for Scientific Databases and Visualization at the Heidelberg Institute for Theoretical Studies.

**Ulrike Wittig** is a postdoc in the group for Scientific Databases and Visualization at the Heidelberg Institute for Theoretical Studies.

**Maja Rey** is a postdoc in the group for Scientific Databases and Visualization at the

**Christian Quast** is a postdoc at Jacobs University Bremen, Germany. He is the project lead of the SILVA project and manages the releases as well as the software development. Additionally, he is heading the implementation of the UniEuk taxonomy framework.

**Janine Felden** is the group leader of the PANGAEA group at the Alfred Wegener Institute–Helmholtz Center for Polar–and Marine Research in Bremerhaven and associated with the MARUM–Center for Marine Environmental Sciences, University of Bremen.

**Frank Oliver Glöckner** is the head of PANGAEA, head of Data at the Computing and Data Center, Alfred Wegener Institute–Helmholtz Center for Polar and Marine Research and MARUM–Center for Marine Environmental Sciences, University of Bremen. He is a professor for Earth System Data Science at the University of Bremen and adjunct professor for Bioinformatics at the Jacobs University Bremen.

**Matthias Lange** is a senior scientist at the IPK-Gatersleben, coordinates the scientific data management and leads the Laboratory Information Management facility within the research group Bioinformatics and Information Technology.

Daniel Arend studied bioinformatics at the Martin Luther University Halle-Wittenberg. He is working as a postdoc at the IPK Gatersleben and his main research focus is on managing, publishing and standardization of research data.

**Sebastian Beier** is a postdoc at the IPK Gatersleben. His main research focus is sequence analysis, especially *de novo* assembly and resequencing studies, and the linking of genotypic and phenotypic data.

**Astrid Junker** is group leader of the research and infrastructure group Acclimation Dynamics and Phenotyping at the IPK Gatersleben and coordinates the plant phenomics infrastructure.

**Uwe Scholz** is the group leader of the research group Bioinformatics and Information Technology at the Leibniz Institute of Plant Genetics and Crop Plant Research (IPK) Gatersleben and coordinates the plant service unit GCBN within de.NBI.

**Danuta Schüler** is the system administrator for the Laboratory Information Management System at the IPK Gatersleben. Her focus is on modeling processes for the LIMS system and data processing, providing user-specific interfaces, web pages for data mapping and user support.

**Hans A. Kestler** is the founding director of the Institute of Medical Systems Biology at the Ulm University and associate group leader at the Leibniz Institute on Aging–Fritz Lipmann Institute Jena.

**Daniel Wibberg** is a postdoc at the Center for Biotechnology of the Bielefeld University and coordinates the de.NBI/ELIXIR-DE training program.

**Alfred Pühler** is a senior research group leader at the Center for Biotechnology of the Bielefeld University and coordinates the German Network for Bioinformatics Infrastructure (de.NBI).

**Sven Twardziok** is a bioinformatician at the Berlin Institute of Health/Charité–Universitätsmedizin Berlin.

**Roland Eils** is the founding director of the Digital Health Center at Berlin Institute of Health/Charité–Universitätsmedizin Berlin and the head of the Health Data Science unit at the Heidelberg University Hospital.

**Juergen Eils** is the group leader of the joint health data groups at the University Clinic of Heidelberg and at Charité Berlin.

**Steve Hoffmann** is a group leader at the Leibniz Institute on Aging and a professor for computational biology at the Friedrich Schiller University Jena. His main research interest is the development of methods to investigate the epigenomic regulation of genome activity.

**Martin Eisenacher** is the coordinator of the research unit Medical Bioinformatics at the Medizinisches Proteom-Center within the Medical Faculty of the Ruhr University Bochum.

**Michael Turewicz** is the head of the group Computational Quantitative Proteomics in the research unit Medical Bioinformatics at the Medizinisches Proteom-Center within the Medical Faculty of the Ruhr University Bochum.
